# Deficiency in Either 4E-BP1 or 4E-BP2 Augments Innate Antiviral Immune Responses

**DOI:** 10.1371/journal.pone.0114854

**Published:** 2014-12-22

**Authors:** Atef Nehdi, Polen Sean, Izzar Linares, Rodney Colina, Maritza Jaramillo, Tommy Alain

**Affiliations:** 1 Department of Biochemistry and Goodman Cancer Center, McGill University, Montreal, Quebec H3A 1A3, Canada; 2 College of medicine, King Saud Bin Abdulaziz University for Health Sciences, King Abdullah International Medical Research Center, King Abdulaziz Medical City, National Guard Health Affairs, P.O. Box 22490, Riyadh 11426, Mail Code 1515, Saudi Arabia; 3 Children's Hospital of Eastern Ontario Research Institute, Department of Biochemistry, Microbiology and Immunology, University of Ottawa, Ottawa, Ontario, Canada; 4 Universidad de la República, Laboratorio de Virología Molecular-PDU, Regional Norte Rivera 1350, CP 50000, Salto, Uruguay; 5 INRS Institut Armand-Frappier Research Centre, 531, boulevard des Prairies, Laval, Quebec, Canada; University of Berne, Switzerland

## Abstract

Genetic deletion of both 4E-BP1 and 4E-BP2 was found to protect cells against viral infections. Here we demonstrate that the individual loss of either 4E-BP1 or 4E-BP2 in mouse embryonic fibroblasts (MEFs) is sufficient to confer viral resistance. shRNA-mediated silencing of 4E-BP1 or 4E-BP2 renders MEFs resistant to viruses, and compared to wild type cells, MEFs knockout for either 4E-BP1 or 4E-BP2 exhibit enhanced translation of *Irf-7* and consequently increased innate immune response to viruses. Accordingly, the replication of vesicular stomatitis virus, encephalomyocarditis virus, influenza virus and Sindbis virus is markedly suppressed in these cells. Importantly, expression of either 4E-BP1 or 4E-BP2 in double knockout or respective single knockout cells diminishes their resistance to viral infection. Our data show that loss of 4E-BP1 or 4E-BP2 potentiates innate antiviral immunity. These results provide further evidence for translational control of innate immunity and support targeting translational effectors as an antiviral strategy.

## Introduction

The 5′ end of all nuclear-transcribed mRNAs possesses a cap structure that is recognized by the eukaryotic translation initiation factor 4E (eIF4E) [1]–[3]. eIF4E binds to the cap as a subunit of a complex (termed eIF4F) containing the large scaffolding protein eIF4G and the RNA helicase eIF4A [3]–[5]. The eIF4F complex facilitates 40S ribosome recruitment and canonical initiation factors, eIF4E and eIF4G, stimulate this reaction [6]. Almost all of the factors involved in the recruitment of the ribosome, including eIF4E, eIF4B, and eIF4G are phosphoproteins whose phosphorylation state correlates with translation efficiency and cellular growth rate [7]. The interaction between eIF4E and eIF4G is regulated by members of the eIF4E-binding proteins (4E-BPs), a family of translational repressors [8]–[10]. The mammalian family consists of three low molecular weight proteins: 4E-BP1, 4E-BP2, and 4E-BP3 [10]. The 4E-BPs compete with eIF4G for a shared binding site on eIF4E [11], [12], in such a way that the binding of 4E-BPs and eIF4G is mutually exclusive [13]. The activity of 4E-BPs is controlled by the mammalian target of rapamycin (mTOR) kinase complex I (mTORC1), which consists of the protein kinase mTOR, RAPTOR (regulatory associated protein of mTOR), GβL (GTPase β-like protein) DEPTOR (disheveled, Egl-10, pleckstrin domain containing mTOR interacting protein) and PRAS40 (proline-rich Akt substrate of 40 kDa) [14]–[16]. Hypophosphorylated 4E-BPs bind with high affinity to eIF4E and repress translation. mTORC1-mediated 4E-BP hyperphosphorylation causes the dissociation of the 4E-BP/eIF4E inhibitory complex and thus stimulates cap-dependent translation [17], [18]. A large body of evidence indicates that the mTOR pathway is an integral part of innate immunity through its critical roles in signaling and translational control of interferon stimulated genes (ISGs) [19]–[22].

Innate immunity constitutes the first line of defence against viral infection and type-I IFN is critical in this process [23]–[28]. Type-I IFNs are synthesized upon the activation of IRF-3 and IRF-7, which act as “master” transcription factors for IFN-α/β mRNAs [22], [29], [30]. Secreted IFN-α/β then activate the Janus kinase (JAK)/signal transducer and activator of transcription (STAT) pathway leading to the transcription of more than one hundred ISGs [31]–[33]. It is well documented that the mTOR pathway is also activated by type-I IFN [34]–[39] and is essential for type-I IFN production and innate immunity [21], [40]. The critical role of the mTOR signaling pathway in innate immunity is based on several findings using MEFs harboring genetic ablation of mTOR-upstream and downstream components. For instance, the lack of the mTOR negative regulator TSC2 in MEFs enhances type-I IFN production [41]. In addition, MEFs knockout for mTOR regulators and effectors (such as AKT, PI3K, 4E-BPs, and S6Ks), have reduced or enhanced (for the 4E-BPs) type-I IFN production [20]–[22], [40], [42]. The mechanism by which lack of both 4E-BP1 and 4E-BP2 leads to the activation of IFN signaling was previously described in 4E-BP1/2 double knockout (DKO) MEFs, and involves translational derepression of *IRF-7* mRNA (a potent transcription factor for type-I IFN genes) [22], Consistent with these results, in PI3K-depleted cells, IRF-7 expression is diminished [20]. These data are further supported by the observation that inhibition of PI3K or mTOR suppresses type-I IFN induction in plasmacytoid dendritic cells (pDCs) and in mice [19], [21], [42].

Based on the knowledge that 4E-BP1/2 DKO in MEFs confers resistance to viral infection, it is conceivable that inhibitors of 4E-BPs could be used as antiviral drugs. As proof of principle, we first asked whether depletion of 4E-BPs by shRNA would confer resistance to virus infection. We thus initiated studies using lentiviruses directed against 4E-BP1 or 4E-BP2 in MEFs. Surprisingly, the single knockdown for either 4E-BP1 or 4E-BP2 alone was sufficient to render MEFs resistant to infection by different viruses. Furthermore, we found that knockout cells for either 4E-BP1 or 4E-BP2 have similar phenotypes, and that re-introduction of one of the missing translational repressors restored susceptibility to virus infection. These results suggest that silencing either 4E-BP1 or 4E-BP2 can protect cells against viral infection and is sufficient to contribute to the translational regulation of innate antiviral responses.

## Results

### Knockdown of 4E-BP1 or 4E-BP2 inhibits VSV replication

**Figure 1:**
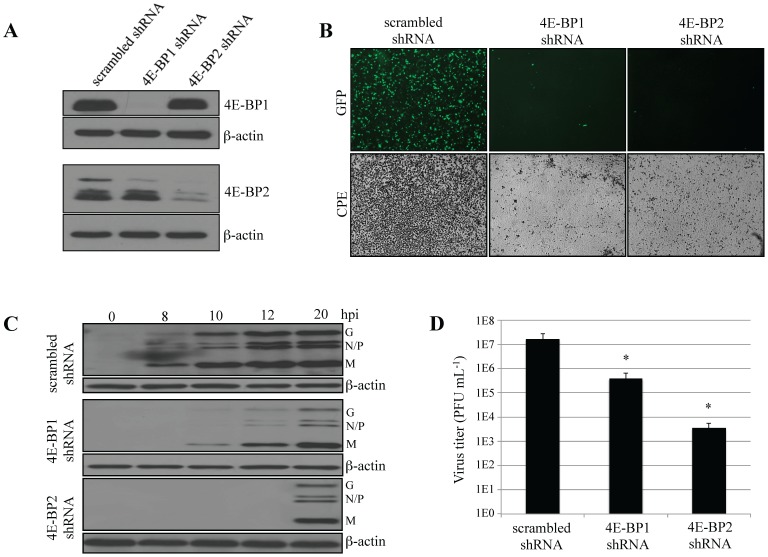
Silencing 4E-BP1 or 4E-BP2 inhibits VSV replication in MEFs. (A) Western blotting analysis of 4E-BP1 or 4E-BP2 expression following transduction of WT MEFs with lentiviruses containing a non-specific shRNA (scrambled), or a shRNA targeting *4E-BP1* or *4E-BP2* mRNA. β-actin served as a loading control. (B) Scrambled MEFs and MEFs knockdown on 4E-BP1 or 4E-BP2 were infected with VSV-GFP at a MOI of 1 PFU/cell and virus replication was assessed by GFP fluorescence and cytopathic effect (CPE). (C) Western blotting analysis for the detection of VSV proteins at the defined time points post-infection with VSV-GFP at a MOI of 1 PFU/cell. β-actin was used as a loading control. (D) Viral titer quantified by plaque assay at 24 hpi with VSV-GFP at a MOI of 1 PFU/cell.

Lentiviruses encoding shRNAs directed against mouse 4E-BP1, 4E-BP2, or a scrambled sequence, were used to lower the expression of 4E-BP1 or 4E-BP2 in WT MEFs. After lentiviral transduction and cell selection, Western blotting analysis was performed to assess the depletion efficiency and specificity of the shRNAs. WT MEFs transduced with lentiviruses expressing shRNA against 4E-BP1 showed little to no detectable 4E-BP1 protein, as compared to MEFs transduced with a scrambled shRNA (Fig. 1A). Lentiviral depletion of 4E-BP1 was specific, since the expression of 4E-BP2 remained unaltered. Similarly, in MEFs transduced with lentiviral particles expressing a shRNA against 4E-BP2, 4E-BP2 was dramatically reduced but 4E-BP1 expression remained unchanged (Fig. 1A). Stable 4E-BP1 or 4E-BP2 knockdown MEFs were then infected with VSV-GFP at a multiplicity of infection (MOI) of 1. WT MEFs transduced with scrambled shRNA showed strong GFP expression and cytopathic effects (CPE) 12 hours post-infection (hpi). Strikingly, MEFs transduced with shRNA against 4E-BP1 or 4E-BP2 showed little to no GFP fluorescence or CPE upon infection (Fig. 1B). Western blotting analysis of the kinetics of viral protein production demonstrated that silencing 4E-BP1 or 4E-BP2 remarkably delayed the appearance of viral proteins (Fig. 1C) and decreased viral yield by up to 50 fold (Fig. 1D). Knockdown of 4E-BP1 or 4E-BP2 in human cells (HeLa) using shRNAs directed against the human proteins led to similar results albeit less pronounced as compared to MEFs (S1 Fig.). Therefore the single silencing of either 4E-BP1 or 4E-BP2 effectively prevents VSV infection/replication.

### Lack of either 4E-BP1 or 4E-BP2 renders MEFs refractory to viral replication

**Figure 2:**
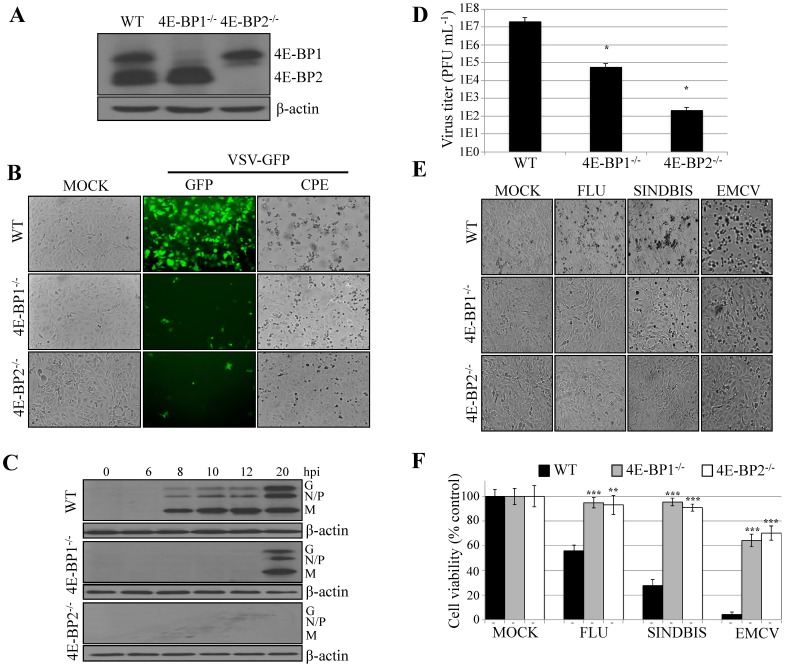
Lack of 4E-BP1 or 4E-BP2 renders MEFs refractory to VSV infection. (A) Western blotting analysis of 4E-BP1 or 4E-BP2 expression in WT, 4E-BP1^−/−^ and 4E-BP2^−/−^ MEFs. β-actin served as a loading control. (B) WT, 4E-BP1^−/−^ and 4E-BP2^−/−^ MEFs were mock-infected or infected at a MOI of 1 PFU per cell with VSV-GFP. Twenty-four hpi, viral infection was assessed by GFP fluorescence and CPE. (C) Western blotting analysis for the detection of VSV proteins at the defined time points post-infection of WT, 4E-BP1^−/−^ and 4E-BP2^−/−^ MEFs with VSV-GFP at a MOI of 1 PFU/cell. β-actin was used as a loading control. (D) Viral titer quantified by plaque assay at 24 hpi in WT, 4E-BP1^−/−^ and 4E-BP2^−/−^ MEFs with VSV-GFP at a MOI of 1 PFU/cell. (E) Photomicrograph of CPE resulting from infections of WT, 4E-BP1^−/−^ and 4E-BP2^−/−^ MEFs with FLU, Sindbis and EMCV virus at 1MOI 12 hpi. (F) Cell viability in experiment in (E) was assessed by MTT assay.

To support the result from the knockdown experiments, we used MEFs derived from 4E-BP1^−/−^ or 4E-BP2^−/−^ knockout mice (Fig. 2A). Wild type (WT), 4E-BP1^−/−^, and 4E-BP2^−/−^ MEFs were infected with VSV WT or VSV-GFP at a MOI of 1. Viral infection was assessed at various times post-infection by fluorescence microscopy and by Western blotting analysis using antibodies against VSV proteins. In WT MEFs, GFP fluorescence was first detected at 12 hpi (Fig. 2B). However, in 4E-BP1^−/−^ and 4E-BP2^−/−^ MEFs, little to no fluorescence was observed at this or later time points post-infection. Western blotting analysis of the infection showed robust expression of VSV proteins in WT MEFs at 8 hpi, but in 4E-BP1^−/−^ MEFs or 4E-BP2^−/−^ MEFs, production of viral proteins was only detectable at 20 hpi (Fig. 2C). Accordingly, VSV-induced CPE was only observed in WT MEFs (Fig. 2B), and cells knockout for either 4E-BP1 or 4E-BP2 showed a reduction of VSV titers by 2 to 5 logs, respectively (Fig. 2D). These data demonstrate that the lack of either 4E-BP1 or 4E-BP2 dramatically impede upon VSV propagation.

To determine if the VSV-resistant phenotype in 4E-BP1^−/−^ and 4E-BP2^−/−^ MEFs is applicable to other RNA viruses, we infected the cells with encephalomyocarditis virus (EMCV), Sindbis virus and influenza virus (FLU). Similar to VSV, cytopathic effects induced by these viruses were severely impaired in 4E-BP1^−/−^ and 4E-BP2 MEFs as compared to their WT counterpart (Fig. 2E). To assess virus-induced cell death, we performed a MTT assay demonstrating higher cell viability for 4E-BP1^−/−^ and 4E-BP2^−/−^ MEFs than for WT MEFs (Fig. 2F). Taken together, these data show that depletion of either 4E-BP1 or 4E-BP2 is sufficient to impair the propagation of a broad spectrum of viruses.

### Cells lacking 4E-BP1 or 4E-BP2 have elevated antiviral cytokine production

**Figure 3:**
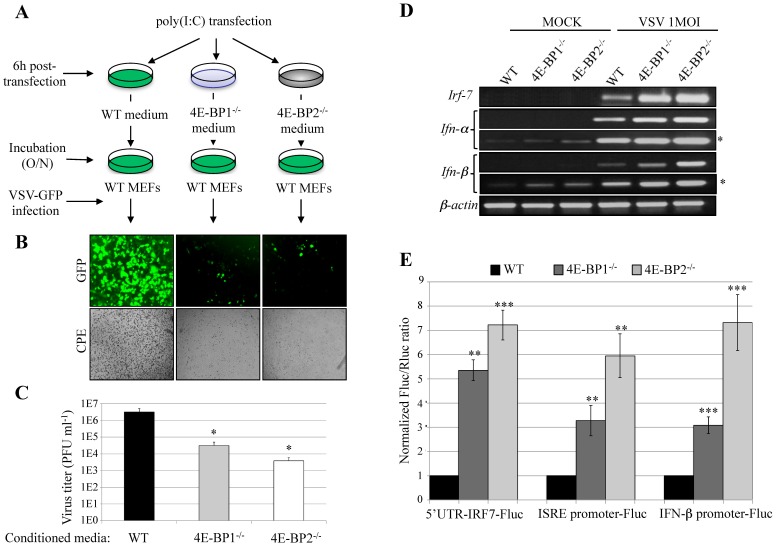
Deficiency of 4E-BP1 or 4E-BP2 enhances type-I interferon production. (A) Protection assay: diagram of experimental protocol: after stimulation with poly(I:C) (6 hours) the medium of WT, 4E-BP1^−/−^ and 4E-BP2^−/−^ MEFs were collected and transferred to three different plates containing WT MEFs. (B) WT MEFs from (A) were then infected with VSV-GFP. The protective effects of the different media were assessed 24 hpi by fluorescence microscopy, cytopathic effect and (C) virus titration. (D) WT, 4E-BP1^−/−^ and 4E-BP2^−/−^ MEFs were infected with VSV at a MOI of 1 PFU/cell for 6 hours and the induction of a type-I IFN response (*Irf-7*, *Ifn-α* and *Ifn*-β mRNA levels) was determined by RT-PCR (*longer exposure). (E) Luciferase assays showing the ratio of expression of the different luciferase constructs harbouring either 5′UTR-IRF-7 (translational level), IFN-α promoter or IFN-β promoter (transcriptional level), normalized to the transfection control (*Renilla* luciferase). Fluc activity/Rluc activity in WT MEFs was set as 1.

To determine whether deletion of either 4E-BP1 or 4E-BP2 increases type-I IFN production, we performed an interferon protection assay (Fig. 3A). Briefly, WT, 4E-BP1^−/−^ or 4E-BP2^−/−^ MEFs were treated for 6 hours with poly(I:C), a synthetic double-stranded RNA and a potent inducer of type-I IFN. The media were then harvested and used to condition WT MEFs. Following overnight incubation with the conditioned media, MEFs were challenged with VSV-GFP (1 MOI) and viral infections were analyzed by fluorescent microscopy and plaque assay (Figs. 3B and C). Culture medium from poly(I:C)-treated WT MEFs was insufficient to protect cells from VSV-GFP infection. In contrast, almost no GFP fluorescence or CPE was observed in WT MEFs incubated with the conditioned media from 4E-BP1^−/−^ or 4E-BP2^−/−^ MEFs (Fig. 3B). Accordingly, virus titers were significantly reduced in cells conditioned with media from MEFs lacking 4E-BP1 (∼2 logs), or 4E-BP2 (∼3 logs) (Fig. 3C). Altogether, these data show that lack of either 4E-BP1 or 4E-BP2 results in cells that secrete cytokines leading to a protective innate immune response.

We next determined the levels of *Ifn-α*, *Ifn-β* and *Irf-7* mRNAs in WT, 4E-BP1^−/−^ and 4E-BP2^−/−^ MEFs by RT-PCR after either mock stimulation, or VSV infection. No *Irf-7* mRNA was observed in mock stimulated cells, whereas the levels of *Ifn-α* and *Ifn-β* mRNAs were higher in MEFs lacking 4E-BP1 or 4E-BP2 as compared to WT MEFs (Fig. 3D). Upon, VSV infection, the induction of *Irf-7*, *Ifn-α*, and *Ifn-β* mRNAs in 4E-BP1^−/−^ and 4E-BP2^−/−^ MEFs was enhanced as compared to WT cells (Fig. 3D). The 5′UTR of *Irf-7* mRNA is highly structured [22], which can be sensitive to changes in the 4E-BPs/eIF4E ratio [8], [43], [44]. To assess the effect of the absence of 4E-BP1 or 4E-BP2 on *Irf-7* mRNA translation, we used a luciferase reporter construct containing the IRF-7 5′UTR [22]. The translation of this mRNA was elevated in MEFs lacking either 4E-BP1 or 4E-BP2 when compared to WT cells (Fig. 3E). Furthermore, to monitor the expression of ISRE and IFN-β genes in 4E-BP1^−/−^ and 4E-BP2^−/−^ MEFs, we used two constructs in which the expression of luciferase is driven by either the ISRE or IFN-β promoter. Luciferase expression from both constructs was increased in 4E-BP1^−/−^ and 4E-BP2^−/−^ MEFs as compared to WT MEFs (Fig. 3E). These results demonstrate that the production of type-I IFN following VSV infection, and the translational regulation of the *Irf-7* mRNA, is enhanced in MEFs lacking either 4E-BP1 or 4E-BP2.

### Re-introduction of 4E-BP1 or 4E-BP2 in double or single knockout MEFs restores the susceptibility to VSV infection

**Figure 4:**
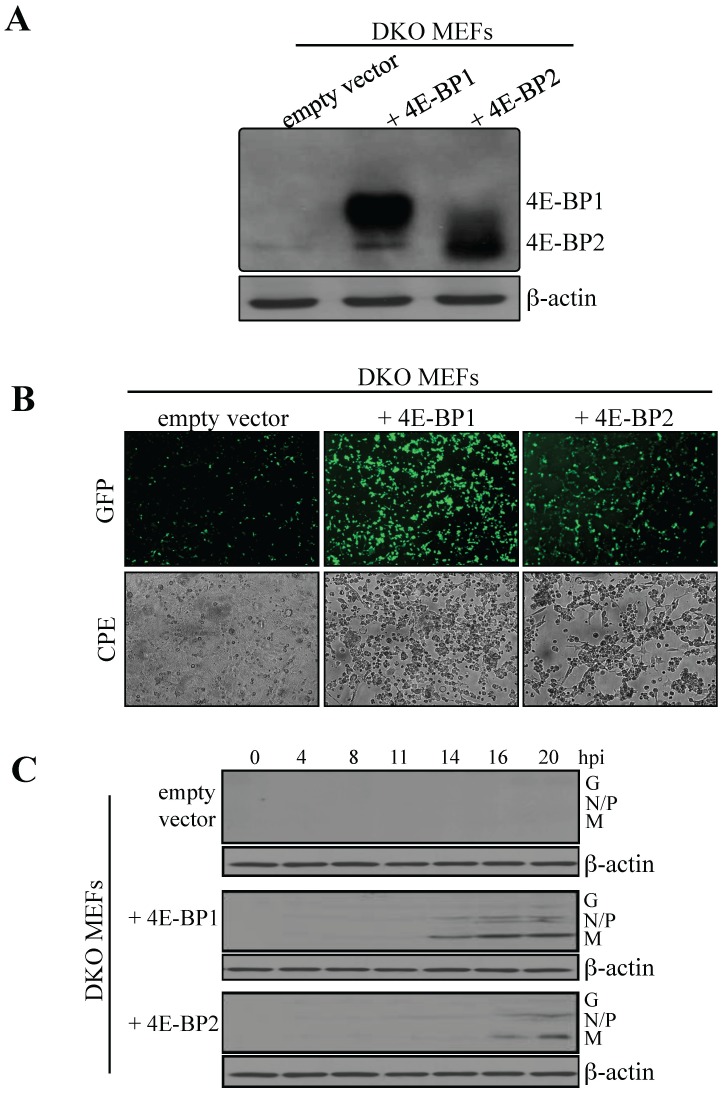
Exogenous expression of 4E-BP1 or 4E-BP2 in MEFs knockout for both translation repressors augments their susceptibility to VSV infection. (A) 4E-BP1/2 DKO MEFs were transduced with retroviruses expressing either 4E-BP1, 4E-BP2 or an empty vector as control. Western blotting analysis for the expression levels of exogenous 4E-BP1 or 4E-BP2 in DKO MEFs. (B) Control and 4E-BP1- or 4E-BP2-expressing DKO MEFs were infected with VSV-GFP at a MOI of 1 PFU/cell for 24 hours and infection was monitored by GFP fluorescence and CPE. (C) Western blotting analysis for the detection of VSV proteins at the defined time points post-infection with VSV-GFP at a MOI of 1 PFU/cell. β-actin was used as a loading control.

**Figure 5:**
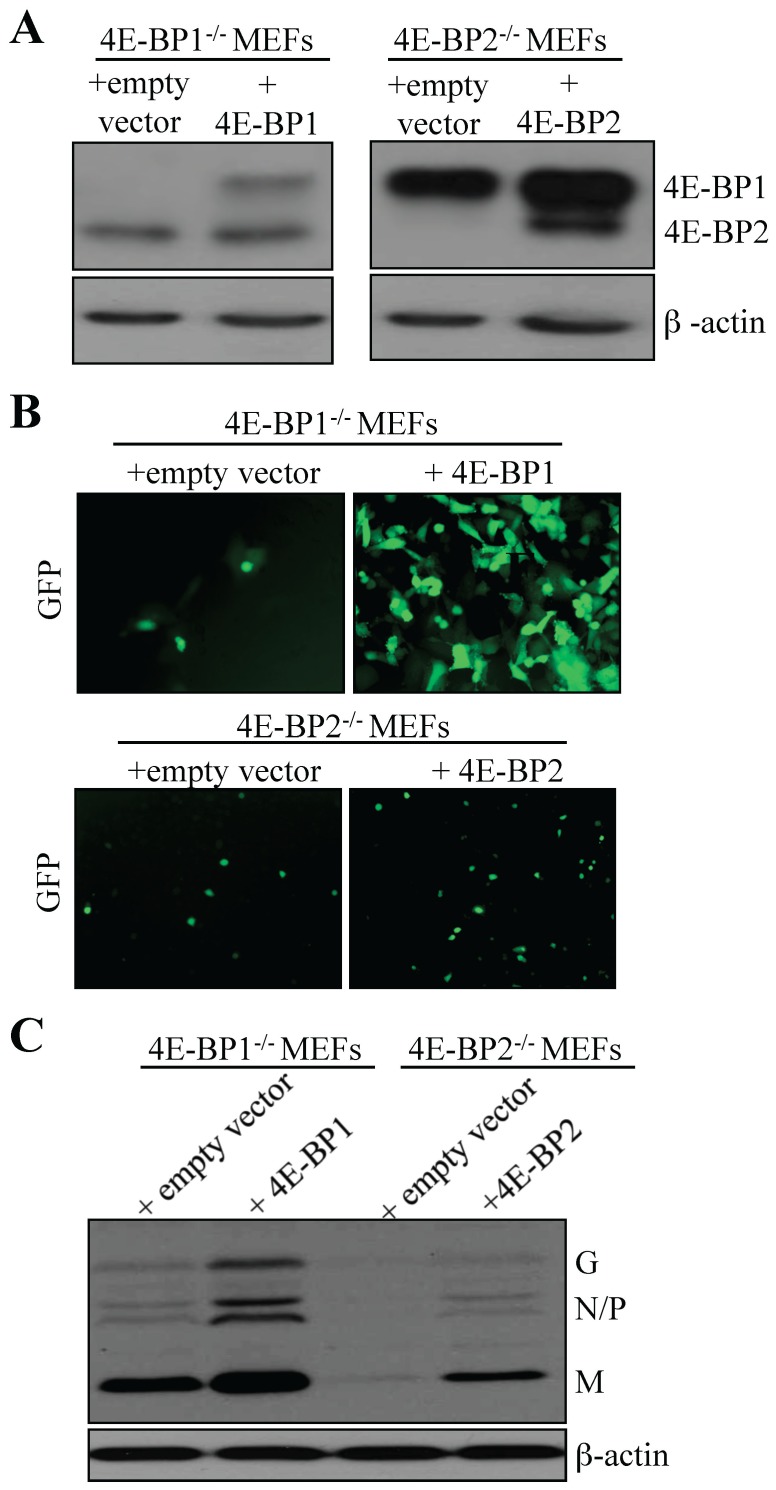
Exogenous expression of 4E-BP1 or 4E-BP2 in respective 4E-BP1^−/−^ or 4E-BP2^−/−^ MEFs increases susceptibility to VSV infection. (A) 4E-BP1^−/−^ and 4E-BP2^−/−^ MEFs were transduced with retroviruses carrying an empty vector (control), or retroviruses expressing either 4E-BP1 or 4E-BP2. Western blotting analysis for exogenous expression of 4E-BP1 and 4E-BP2 in their respective single knockout MEFs. (B) Control and transduced MEFs were infected with VSV-GFP at a MOI of 1 PFU/cell and viral infection was assessed by GFP fluorescence and by (C) Western blotting analysis for VSV viral protein expression.

To support the findings that lack of either 4E-BP1 or 4E-BP2 is sufficient to confer resistance to viral infection, we reintroduced 4E-BP1 or 4E-BP2 in 4E-BP1^−/−^4E-BP2^−/−^ DKO MEFs. DKO MEFs were transduced with a control retrovirus, or a retrovirus expressing either recombinant 4E-BP1 or 4E-BP2. Expression of 4E-BP1 or 4E-BP2 was confirmed by Western blotting (Fig. 4A). Cells were then infected with VSV-GFP at a MOI of 1 PFU/cell and infection was analyzed by fluorescence microscopy at 20 hpi. Re-expression of either 4E-BP1 or 4E-BP2 augmented the susceptibility of DKO MEFs to VSV infection (Fig. 4B). Viral proteins appeared after 14 hpi in DKO MEFs expressing recombinant 4E-BP1, and at 16 hpi in DKO MEFs expressing recombinant 4E-BP2. By contrast, in control cells transduced with empty vector, viral proteins were not detected (Fig. 4C). Using the same strategy, 4E-BP1 or 4E-BP2 were reintroduced into 4E-BP1^−/−^ and 4E-BP2^−/−^ MEFs, respectively After cell selection, recombinant 4E-BP expression was assessed via Western blotting (Fig. 5A) and cells were infected with VSV-GFP at a MOI of 1 PFU per cell. At 16 hpi, an increase in infection was found rescued 4E-BP1^−/−^ MEFs compared to control cells; however, little differences in terms of fluorescence were detected between control and rescued 4E-BP2^−/−^ MEFs at this time or later time points (Fig. 5B). Western blotting analysis of whole extracts prepared from infected cells (16 hpi) confirmed the difference in infection observed by fluorescence between 4E-BP1^−/−^ MEFs and cells rescued with 4E-BP1 (Fig. 5C). There was also an increase in viral protein accumulation in rescued 4E-BP2^−/−^ MEFs compared to control cells when assessed by Western blotting although less dramatic than in 4E-BP1^−/−^ rescued cells (Fig. 5C). Altogether, these results indicate that the reintroduction of either 4E-BP1 or 4E-BP2 in cells lacking these translation repressors enhance their susceptibility to VSV infection. The data from knockdown on 4E-BP1 or 4E-BP2 expression in WT MEFs, and the restored susceptibility of 4E-BP1/2 DKO MEFs following reintroduction of either 4E-BP1 or 4E-BP2, provide genetic evidence that the virus resistant phenotype of 4E-BP1^−/−^ and 4E-BP2^−/−^ MEFs is associated with 4E-BP1 or 4E-BP2 expression.

## Discussion

Our results show that MEFs lacking 4E-BP1 or 4E-BP2 have enhanced type-I IFN production, higher resistance to viral infection, and increased IRF-7 5′UTR-reporter gene translation as compared to WT cells. Furthermore, re-introduction of one of the missing translational repressors in DKO (4E-BP1^−/−^ 4E-BP2^−/−^) MEFs, or single knockout MEFs, renders cells increasingly susceptible to viral infection. Our data indicate that the loss of one 4E-BP is sufficient to alleviate a negative regulatory effect on the translation of the *Irf-7* mRNA, potentiating a more efficient innate immune response. Pathogens, including viruses, induce an antimicrobial and pro-inflammatory cytokine production that serves as the initial barrier to infection [22], [26]–[28], [45]. However, continued cytokine expression can have detrimental effects; therefore, the balance between activators and repressors of innate immune responses needs to be tightly regulated [46]–[49]. Many activators of the type-I IFN have been described, and recently repressors of this signaling pathway have been reported. The SOCS (suppressors of cytokine signaling) are classical examples [50]. Our study strengthens the findings that 4E-BP1 and 4E-BP2 act as translation repressors of innate immunity by negatively regulating mRNA translation of the master regulator of type-I IFN, IRF-7 [22].

In mammalian cells, there are three 4E-BPs (4E-BP1, 4E-BP2, and 4E-BP3), with 4E-BP1 being the best characterized [9], [10]. In MEFs, 4E-BP3 is expressed at undetectable levels [22], [51]. MEFs lacking both 4E-BP1 and 4E-BP2 are extremely resistant to viral infections due to increased type-I IFN production and high *Irf-7* mRNA translation [22]. Kaur and colleagues previously reported that cells lacking only 4E-BP1 have increased type-I IFN production [41]. Here we show that silencing either 4E-BP1 or 4E-BP2 augments the cell resistance to viral infections in MEFs. We found that loss of 4E-BP1 or 4E-BP2 results in reduced translational repression of *IRF-7* and an enhancement of innate antiviral responses. While we observed variations in viral susceptibility between cells expressing either 4E-BP1 or 4E-BP2, the significance of these differences is currently being investigated to determine whether distinct ISGs could be selectively regulated at the translation level by either 4E-BP1 or 4E-BP2. Interestingly, 4E-BP expression levels vary among different tissues [52]. Such a definite expression of 4E-BPs may suggest potentially distinct biological functions with regard to tissue-specific gene expression, cell differentiation, and perhaps innate immune responses [53], [54].

In conclusion, we report that cells lacking 4E-BP1 or 4E-BP2 have an augmented type-I IFN production and are resistant to various viral infections. These results confirm that 4E-BP1 and 4E-BP2 play critical roles in the translation regulation of innate immune responses and support the basis for targeting these translational repressors as an antiviral strategy.

## Materials and Methods

### Cell lines and Viruses

MEFs derived from WT, 4E-BP1^−/−^, 4E-BP2^−/−^ and 4E-BP1^−/−^/4E-BP2^−/−^ mice (provided by N. Sonenberg, McGill University) were immortalized by sequential passaging [22], [55]. All cell lines were cultured in DMEM +10% FBS. Vesicular stomatitis virus (VSV; Indiana strain), or recombinant VSV harboring a green fluorescent protein (GFP) transgene (VSV-GFP; gift of John Hiscott, VGTI Institute, Miami Florida). Influenza virus A/HK/1/68-MA20 was provided by E. G. Brown (University of Ottawa), EMCV K-2 by V. Agol (Russian Academy of Medical Sciences), and Sindbis virus by J. Berlanga (Universidad Autonoma de Madrid). EMCV, Sindbis virus and VSV were propagated in BHK21 cells. Infections were performed at a multiplicity of infection (MOI) of 1 PFU/cell or as indicated. Specifically, virus was diluted in serum-free DMEM and added to cells for 1 to 2 hours to allow adsorption. Virus was then removed, and DMEM with 10% (FBS) was added for the remaining time. Virus titers were determined by a standard plaque assay.

### Western blotting analysis

MEFs were homogenized in buffer A (50 mM Tris-HCl, pH 7.4, 100 mM NaCl, 1% Triton X-100, 1 mM EDTA, 1 mM dithiothreitol, protease inhibitor cocktail (Roche), 20 mM β-glycerophosphate, 0.25 mM Na_3_VO_4_, 10 mM NaF, 10 nM okadaic acid, 1 mM PMSF) and incubated for 30 min at 4°C. Cell debris was removed by centrifugation at 10,000 *g* for 10 min at 4°C and total protein content was determined using a Bio-Rad assay. Laemmli sample buffer was added to the supernatant, which was then subjected to SDS–PAGE (15%). Proteins were transferred onto a nitrocellulose membrane, which was blocked for 2 hours at room temperature with 5% skim milk in PBS containing 0.2% Tween-20 (PBS-T) and washed twice with PBS-T. The membrane was incubated overnight at 4°C with primary antibodies followed by three 10-min washes in PBS-T and further incubated with peroxidase-coupled secondary antibody for 30 min at room temperature, and washed three times. Detection of peroxidase-coupled secondary antibody was performed with ECL (GE-Healthcare). 4E-BP1 and 4E-BP2 antibodies were purchased from Cell Signaling and the β-actin antibody was purchased from Santa Cruz Biotechnology. VSV antibody was a gift from J. Bell.

### Rescue experiments

pBABE-4E-BP1, pBABE-4E-BP2 and empty vector constructs (pBABE-puro) were transfected into phoenix-293-T packaging cells using Lipofectamine 2000 (Invitrogen) according to the manufacturer's instructions. After 48 h, virus-containing medium was filtered, collected and used to infect *4E-BP1* ^−/−^ *4E-BP2* ^−/−^ MEFs, *4E-BP1* ^−/−^ MEFs and *4E-BP2* ^−/−^ MEFs in the presence of 5 mg ml^-1^ of polybrene (Sigma-Aldrich). Cells were re-infected the next day and supplemented with puromycin (5 µg ml^-1^, Sigma-Aldrich) to be selected for five days. The expression of the exogenous 4E-BPs was confirmed by Western blotting.

### shRNA against 4E-BP1 and 4E-BP2

Five different shRNA were used in this study:

Control shRNA is a non-target shRNA: (sigma: TRC1/1.5) CCGGCAACAAGATGAAGAGCACCAACTC- GAGTTGGTGCTCTTCATCTTGTTGTTTTT

shRNA against human *4E-BP1*: (hshBP1)(sigma: TRCN0000040203): CCGGGCCAGGCCTTATGAAAGTGATCTCGAGATCACTTTCATAAGGCCTGGCTTTTTG

shRNA against human *4E-B2*: (hshBP2)(sigma: TRCN0000117814) CCGGCGCAGCTACCTCATGACTATTCTCGAGAATAGTCATGAGGTAGCTGCGTTTTTG

shRNA against mouse *4E-BP1*: (mshBP1)(sigma: TRCN0000075612): CCGGAGGCGGTGAAGAGTCACAATTCTCGAGAATTGTGACTCTTCACCGCCTTTTTTG

shRNA against mouse *4E-BP2*: (mshBP2)(sigma: TRCN0000075614) CCGGCGCCTTAATTGAAGACTCCAACTCGAGTTGGAGTCTTCAATTAAGGCGTTTTTG

PLKO.1-puro vector containing the appropriate shRNA, was transfected into HEK-293-T packaging cells as described by Sigma Aldrich (http://www.sigmaaldrich.com/life-science/functional-genomics-and-rnai/shrna/trc-shrna-products/lentiviral-packaging-mix.html). After 48 hours, medium containing lentivirus particles was filtered, collected and used to infect WT MEFs or HeLa cells. Forty-eight hpi, viral particle-containing medium was removed and replaced with fresh medium containing 5 µg/ml (for MEFs)/2 µg/ml (for HeLa) of puromycin (Sigma-Aldrich) for selection of transduced cells for five days.

### RT–PCR and RNA extraction

Total RNA was extracted using Trizol reagent (Invitrogen) according to the manufacturer's instructions. Total RNA (1 µg) was reverse transcribed (RT) with Superscript III reverse transcriptase (Invitrogen) for 1 hour at 50°C using oligo dT. 1 µl of RT template was incubated with specific primers (described below) and with Taq Polymerase (Fermentas) according to the supplier's instructions. The number of PCR cycles ranged from 23 to 34 depending on the linearity of the reaction. PCR primers included (5′ to 3′): mIFN-α sense (CCTTCCACAGGATCACTGTGTACCT), IFN-α antisense (TTCTGCTCTGACCACCTCCC); mIFN-β sense (CACAGCCCTCTCCATCAACT), mIFN-β antisense (TCCCACGTCAATCTTTCCTC); mIRF-7 sense (ATGATGGTCACATCCAGGAACCCA), mIRF-7 antisense (TCAGGTCTGCAGTACAGCCACAT); mβ-actin sense (GGACTCCT ATGTGGGTGACGAGG), mβ-actin antisense (GGGAGAGCATAGCCCTCGTAGAT). PCR reactions were optimized to measure the exponential phase on the amplification curve.

### Virus titration by plaque assay

The day prior to the assay, HEK (human embryonic kidney) cells were seeded onto six-well plates at 3×10^5^ cells/well in 2 ml of growth media. Cells were maintained at 37°C with 5% CO_2_. On the day of assay, serial viral dilutions were made in the inoculation medium. Growth medium was aspirated from cells, and 0.5 ml of each dilution was added per well and allowed to adsorb for 2 hours at 37°C in a humidified incubator containing 5% CO_2_. The agarose overlay was prepared by mixing one part of 2×McCoy's 5A containing 30% heat-inactivated FBS and 2% DMSO with one part of pre-warmed 1.5% Sea Plaque low melting agarose. The mixture was kept warm at 42°C. The inoculum was removed and 5 ml of the pre-warmed agarose overlay was added to the cells. The agarose was allowed to set for at least 30 min at room temperature prior to a 35 or 37°C incubation with 5% CO_2_. Plaques were counted on day 5 as follows: 3 ml of 10% formaldehyde was added to each well, the plates were allowed to sit at room temperature for 1 h, and then the formaldehyde was aspirated and agarose overlay was carefully removed. The cells were stained with 2 ml of 0.4% crystal violet in an aqueous alcohol solution (Becton Dickinson Microbiology Systems, Cockeysville, MD) for 2 min. The staining solution was decanted and the plaques were washed with water before inverting the plates and allowing them to dry. The plaques were counted and expressed as plaque forming units (PFU). Results are the mean ±SD of three independent experiments, the P values were calculated using the Student's T-test where: (*) P<0.05; (**) P<0.01; (***) P<0.001.

### Plasmid constructs and luciferase assay

MEFs were seeded at 3×10^4^ cells per 24-well then were transiently co-transfected with 500 ng of 5′ UTR-IRF-7-Fluc vector and 50 ng of transfection control, Renilla luciferase vector (Rluc; Promega), which is under the control of the CMV promoter, using Lipofectamine 2000 as describedhttp://www.nature.com/nature/journal/v452/n7185/full/nature06730.html - B36 in [22]. For the promoter reporter assay, we used the same condition as described previously; MEFs were co-transfected with a plasmid containing either the IFN-α promoter or the IFN-β promoter followed by firefly luciferase (FL). In this last assay we used as a transfection-control, a plasmid containing the thymidine kinase (TK)-promoter followed by Renilla luciferase RL (kind gift from D. Muruve). Twenty hours after transfection, cell extracts were prepared in passive lysis buffer (Promega) and assayed for Rluc and Fluc activity in a Lumat LB9507 bioluminometer (EG&G Bertold) using a dual-luciferase reporter assay system (Promega), according to the manufacturer's instructions. Fluc activity was normalized against Rluc activity, which was used as a transfection control. Data represent the mean ±SD of firefly luciferase value of three to six samples, normalized by *Renilla* luciferase activity. The P values were calculated using the Student's T-test where: (*) P<0.05; (**) P<0.01; (***) P<0.001.

### Protection assay

The VSV protection assay was performed as followed: WT, 4E-BP1^−/−^ and 4E-BP2^−/−^ MEFs, were either mock-treated, or transfected with 1 µg/ml of poly(I:C) (Sigma) using FuGENE 6 transfection reagent (Roche) according to the manufacturer's protocol. Supernatants were collected at 6 hours from mock or stimulated cells and were subsequently added to the cells overnight. On the next day, cells were infected with VSV WT or VSV-GFP at a MOI of 1 for a 24-hour period. Viral infection was assessed by fluorescence microscopy and cell viability was measured at 12 or 24 hours post-infection by 3-(4,5-dimethylthiazol-2-yl)-2,5-diphenyl-2*H*-tetrazoium bromide (MTT) assay.

### MTT cell viability assay

MTT (3-(4,5-dimethylthiazol-2-yl)-2,5-diphenyl tetrazolium bromide) (Sigma, St. Louis, MO, USA) was used to assess cell viability after viral infection. MTT was diluted to 5 mg/ml in sterile phosphate buffered saline (PBS) and filtered once through a 0.45 µm filter. The diluted MTT was added to the wells in a final volume of 25 µl/well. Four hours later, 100 µl of DMSO were added to each well. The microtiter plates were incubated at 37°C overnight to dissolve reduced MTT crystals. Absorbance values were determined by ELISA reader (Multiskan Plus, Lab Systems, Helsinki, Finland) at dual wavelengths of 570 and 690 nm. Results are the mean ±SD of three independent experiments, the P values were calculated using the Student's T-test where: (*) P<0.05; (**) P<0.01; (***) P<0.001.

## Supporting Information

S1 Fig Knockdown of 4E-BPs in HeLa cells, (A) Fluorescent microscopy of HeLa cells infected with VSV-GFP. (B) Western blotting analysis showing the kinetics of VSV protein expression following 4E-BP knockdown.
(TIFF)
