# Supplementary figures and images for: Deficiency in Either 4E-BP1 or 4E-BP2 Augments Innate Antiviral Immune Responses
Source: PLoS One. 2014 Dec 22;9(12):e114854. doi: 10.1371/journal.pone.0114854 (PMC4273997; doi:10.1371/journal.pone.0114854)

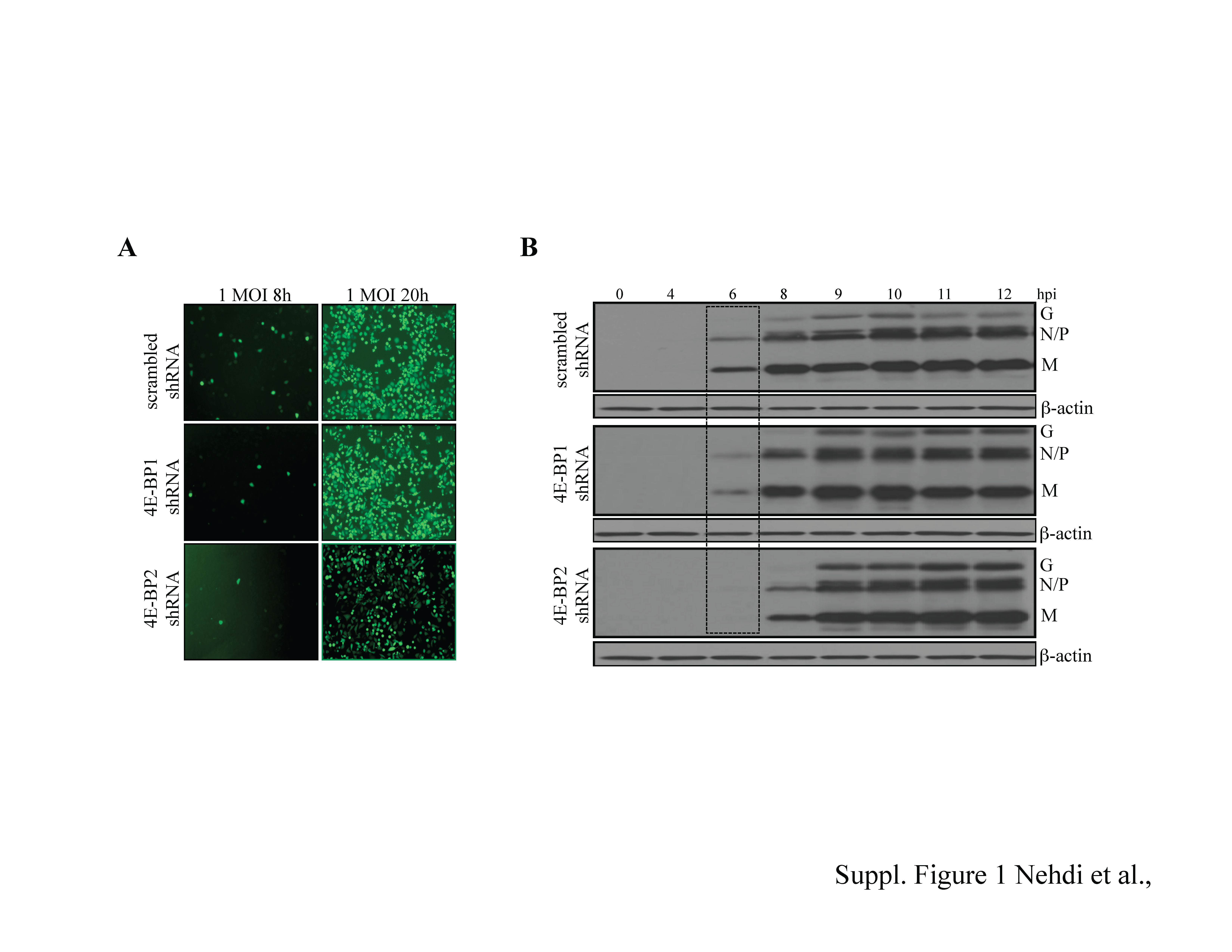

Supplement: S1 Fig — Knockdown of 4E-BPs in HeLa cells, (A) Fluorescent microscopy of HeLa cells infected with VSV-GFP. (B) Western blotting analysis showing the kinetics of VSV protein expression following 4E-BP knockdown. (TIFF) [file pone.0114854.s001.tiff]
